# Association between a family history of cancer and multiple primary lung cancer risks: a population-based analysis from China

**DOI:** 10.1186/s12890-023-02676-1

**Published:** 2023-10-31

**Authors:** Chen-Hui Ni, Mu-Ting Wang, Yan-Qi Lu, Wei Zheng, Chun Chen, Bin Zheng

**Affiliations:** 1 Department of Thoracic Surgery, The Affiliated Union Hospital, Fujian Medical University, No.29 Xinquan Road, Fuzhou, 350001 Fujian Province China; 2 Key Laboratory of Cardio-Thoracic Surgery, Fujian Medical University, Fujian Province University, Fuzhou, 350001 Fujian China

## Abstract

**Objectives:**

The incidence of multiple primary lung cancer (MPLC) has increased in recent years. The risk factors of MPLC are not well studied, especially in the Asian population. This case-control study investigated the association between a family history of cancer and MPLC risk.

**Methods:**

We used data from people who surgically confirmed MPLC with at least 2 nodes of Fujian Medical University Union Hospital and matched 1:2 normal individuals as controls between 2016 and 2017. Information on age, sex, lifestyle, personal history, and family history of cancer was collected using a self-administered questionnaire, and odds ratios (OR) were estimated using unconditional logistic regression.

**Results:**

We included 2 104 patients. In total, 321 patients with histologically confirmed MPLC and 642 healthy controls were studied. The significantly higher ratio of current smokers was observed for the cases than the controls (54.1% vs. 30.0%). A family history of LC in first-degree relatives of the cases reported a significantly higher proportion than in the controls (15.3% vs. 8.6%). Family history of all cancers and LC significantly increased the risk of MPLC (OR = 1.64, *P* = 0.009 and OR = 2.59, *P* = 0.000, respectively). The multivariate analysis identified a significantly increased risk of MPLC (OR = 2.45, *P* = 0.000) associated with parents and siblings influenced by LC history. The younger age (aged < 55 years) of LC cases at diagnosis exhibited a significantly increased risk of MPLC (OR = 2.39, *P* = 0.000). A significant association with a family history of LC was found for male squamous carcinoma and male adenocarcinoma (OR = 1.59, p = 0.037 and OR = 1.64, p = 0.032, respectively). A positive association with LC history was only observed for female adenocarcinoma (OR = 2.23, p = 0.028). The risk of MPLC was not significantly associated with A family history of cancers in non-smokers (OR = 0.91, *P* = 0.236). Ever-smokers with a positive family history of cancer or LC had a significantly elevated risk of MPLC (OR = 4.01, *P* = 0.000 and OR = 6.49, *P* = 0.000, respectively). We also observed a very elevated risk for smokers with no family history (OR = 3.49, *P* = 0.000). Such a positive association was also observed in ever-smokers with no family history of LC (OR = 3.55, *P* = 0.000). Adenocarcinoma in females was prevalent and significantly associated with a family history of LC in risk of MPLC compared with other histologic subtypes.

**Conclusions:**

Our findings suggest an association between a family history of LC and MPLC risk among an Asian population. Smoking status and family history of LC have a synergistic effect on MPLC. These findings indicate that MPLC exhibits familiar aggregation and that inherited genetic susceptibility may contribute to the development of MPLC.

## Introduction

Lung cancer (LC) is one of the most prevalent cancers with a high global disease burden [1]. The incidence of multiple nodules is increasing clinically, and the incidence of MPLC is 1-7% [2, 3]. The risk factors for MPLC are currently unclear. Each subtype of LC has different risk factors [4, 5]. Undoubtedly, cigarette smoking is one of the leading causes of LC. Tobacco can increase LC risk by 5-20% compared with never-smokers in both males and females, even though females smoking prevalence is only approximately 4% [6]. MPLC has different diagnostic criteria and prognoses compared with solitary primary LC [7]. Therefore, it is essential to verify potential risks other than tobacco to clarify the high LC prevalence in China.

Familial aggregation may be one of the factors causing cancers because of genetic inheritance or other unidentified mechanisms [8, 9]. This finding is also supported using the segregation analysis of LC families [10]. Studies and meta-analyses have provided additional evidence of familial aspects of LC risk [11–13]. However, the association between a family history of cancer and MPLC has not been well evaluated, especially in Chinese populations.

The present case-control study investigated familial aggregation of MPLC in the Chinese population using data from 320 MPLC cases and 640 controls. We also included potential risk factors, such as smoking, tobacco smoke exposure, and histology.

## Materials and methods

### Population

A total of 320 patients with newly diagnosed and histologically confirmed multiple primary lung cancer (MPLC) patients were treated at Fujian Medical University Union Hospital in 2016–2017. Based on previous reports, the following criteria were used to categorize MPLC. Tumors with different histologies or subtypes, or tumors with the same histology but no distant or mediastinal lymph node metastasis and distinct molecular genetic characteristics.

Synchronous MPLC were defined as those that underwent resection or treatment simultaneously within 2 years after the initial surgery, and metachronous MPLC were defined if the second LC was treated separately more than 2 years after the initial surgery. Patients with neoadjuvant therapy and history of malignant tumor were excluded. Pathological diagnosis of small cell lung cancer was also ruled out.

As controls, 640 healthy individuals were randomly selected from the outpatient clinic providing general medical care to the communities in the study area. The controls were matched to LC cases (2:1 ratio) based on age (± 5 years) and area of living (rural/urban).

### Collection of information

After obtaining verbal consent, all cases and controls were interviewed or telephoned by a trained interviewer in the hospital (cases) or outpatient department (controls). We applied a structured questionnaire including information (among other items) on demographic characteristics, tobacco and passive smoking history, and family history of cancer in first-degree relatives. Patients with a history of cancer or severe respiratory disease were excluded. Incomplete information on first-degree family history and missing data for other variables were also excluded.

Environmental tobacco smoke was assessed by asking the question about domestic exposure more than once per week and/or smoke exposure at work. Passive smoking includes exposure to cigarette smoke and vapors from cigars, hookah, marijuana, and even e-cigarettes. Continuous and cumulative smoking of at least 100 cigarettes never constitutes smokers. Ex-smokers were defined as those who had quit smoking at least 3 years before the interview.

This study was approved by the ethics committee of the Fujian Medical University Union Hospital.

### Statistical analysis

The descriptive statistics included medians and ranges for continuous variables and percentages for categorical variables, which were compared using Wilcoxon and χ2 tests, respectively. Univariate and multivariate analyses for prognostic factors were conducted using Cox proportional hazards regression models to estimate hazard ratios and 95% Cis. Logistic regression analysis was used to calculate odds ratios and 95% CIs and to investigate significant factors associated with MPLC. All data were analyzed using SPSS Statistics version 23 (IBM, Chicago, IL, USA). A *P*-value of <0.05 was considered statistically significant.

## Results

### Characteristics of cases and controls

The demographic characteristics of the subjects in this study was shown at Table 1. The cases and controls were matched for age and sex. No differences were found between the cases and controls with regard to age, sex, occupation, dwelling, diabetes, history of respiratory disease, education, body mass index, and alcohol consumption.

**Table 1:** Demographic characteristics of cases and controls

Factors	Cases n = 320 (%)	Controls n = 640 (%)	*P* value
Age			
Mean age	59.1	58.8	0.447
<55 years	115(36.0)	222(34.7)	
≥55 years	205(64.1)	418(65.3)	0.702
Sex			
Male	128(40.0)	256(40.0)	
Female	192(60.0)	384(60.0)	1.000
Occupation			
Office work	66(20.6)	141(22.0)	
Industrial work	88(27.5)	166(26)	
Agriculture or forestry	60(18.8)	115(18.8)	
Others	106(33.1)	218(34.1)	0.914
Dwelling			
City	221(69.1)	440(68.9)	
Countryside	99(31.0)	200(31.2)	0.921
Diabetes			
Yes	39(12.2)	70(11.0)	
No	281(87.8)	570(89.1)	0.565
History of respiratory disease			
Yes	48(15.0)	77(12.0)	
No	272(85.0)	563(88.0)	0.198
Education			
No formal education No formal education No formal education No formal education	59(18.4)	91(14.2)	
<6 years ≤ 6 years < ≤ 6 years	92(29.4)	197(30.8)	
Secondary	73(22.8)	153(24.0)	
University	96(30.0)	199(31.1)	0.405
Body Mass Index (BMI)			
<18	101(31.6)	188(29.3)	
18–24	129(40.3)	268(41.9)	
>24	90(28.1)	184(28.8)	0.610
Alcohol drinking			
Yes 2882()	32(10.0)	63(9.9)	
No	288(90.0)	577(90.1)	0.939

Table 2 shows the smoking habits of the cases and controls. The proportion of non-smokers was obviously lower among the case group than the control group (26.9% vs. 51.9%). An evidently higher ratio of current smokers was observed for the cases than the controls (54.1% vs. 30.0%). Mean years smoked in the case group were also evidently higher than the control group. Furthermore, pack-years of smoking and environmental tobacco smoke (ETS) exposure were obviously higher for the cases than the controls. There were no differences in cigarette type between the cases and controls.

**Table 2:** Characteristics of cases and controls according to smoking habits

Factors	Cases n = 320 (%)	Controls n = 640 (%)	*P* value
Smoking			
Never-smokers	86(26.9)	332(51.9)	
Ex-smokers	61(19.1)	116(18.1)	
Current smokers	173(54.1)	192(30.0)	0.000
Mean years smoked			
Never-smokers			
Ex-smokers	26.4	18.7	
Current smokers	34.8	29.8	0.000
Pack-years			
0	86(26.9)	332(51.9)	
1–20	60(18.8)	206(32.2)	
21–30	57(17.8)	50(7.8)	
≥31	117(36.6)	52(8.1)	0.000
Type of cigarettes			
Filter	191(81.6)	254(82.4)	
Non-filter	43(18.4)	54(17.5)	
Both	26(11.1)	28(9.2)	0.572
Environmental tobacco smoke (ETS) exposure			
Absent	99(30.9)	229(35.8)	
Present	221(69.1)	411(64.2)	0.000

Table 3 describes the characteristics of the family members for each groups. No significant differences were observed in the mean age of first-degree relatives (parents and siblings) between the two groups. The number of siblings and the mean number of siblings were equally distributed between the studied groups. The first-degree relatives of cases revealed a evidently higher proportion of smoking habits than the controls. Mean years smoked among fathers and siblings of the cases were obviously higher than those of the controls. Risk of MPLC.

**Table 3:** Characteristics of cases and controls according to families

Factors	Cases n = 320 (%)	Controls n = 640 (%)	*P* value
Mean age			
Father	80.1	79.8	0.218
Mother	79.2	78.9	0.198
Siblings	60.0	59.0	0.441
Smoking of relatives			
Father	218(68.1)	379(59.2)	0.009
Mother	42(13.1)	67(10.5)	0.000
Siblings	424(49.1)	737(41.1)	0.000
Mean years smoked Mean years smoked Mean years smoked Mean years smoked			
Father	40.4	37.8	0.000
Mother	30.1	29.7	0.083
Siblings	24.5	23.4	0.000
Number of siblings			
0–1	86(26.9)	166(25.9)	
2–4	172(53.8)	345(53.9)	
>4	62(19.4)	129(20.2)	0.871
Mean number of siblings	2.7	2.8	0.088

Table 4 shows that a evidently higher proportion of cases reported a family history of LC in first-degree relatives of the cases than in the controls (15.3% vs. 8.6%). The same results were observed in the history of all cancers. LC was reported in 29 fathers of the cases and 33 fathers of the controls (9.1% vs. 5.2%, *P* = 0.000), 7 mothers of the cases and 7 of mothers of the controls (2.1% vs. 1.1%, *P* = 0.000), 20 siblings of the cases, and 19 of siblings of the controls (6.2% vs. 3.0%, *P* = 0.0000).

**Table 4:** Odds ratios (OR) of MPLC associated with family history of cancer in a first-degree relative

Family history	Cases n = 320	Controls n = 640	Crude OR (95% CI)	*p*	Adjusted^a^ OR (95% CI)	*p*
Family history of cancer						
None	219(68.4)	525(82.2)	1.00		1.00	
Yes	101(31.6)	115(17.8)	1.92(1.51–2.42)	0.000	1.64(1.26–2.08)	0.009
Family history of LC						
None	271(84.7)	585(91.4)	1.00		1.00	
Yes	49(15.3)	55(8.6)	2.87(2.03–4.16)	0.000	2.59(1.87–3.77)	0.000
Age of onset with LC						
≥55 years	31	42	1.00		1.00	
<55 years	18	15	2.88(1.91–3.99)	0.000	2.39(1.61–3.79)	0.000
First-degree relative with LC						
Father						
No	291	607	1.00		1.00	
Yes	29	33	1.81(1.37–2.38)	0.000	1.55(1.02-2.00)	0.013
Mother						
No	313	633	1.00		1.00	
Yes	7	7	2.74(2.14–4.67)	0.000	2.45(1.00-2.99)	0.000
Siblings						
No	299	620	1.00		1.00	
Yes	20	19	1.84(1.30–2.64)	0.000	1.66(1.11–2.54)	0.001
Family history of breast cancer						
No	306	625	1.00		1.00	
Yes	14	15	1.54(1.18–2.13)	0.000	1.49(1.10-2.00)	0.042
Family history of gastrointestinal cancer						
No	245	566	1.00		1.00	
Yes	75	74	1.01(0.84–1.21)	0.000	0.97(0.80–1.18)	0.291

^a^Adjusted for age, dwelling, education, ETS exposure, BMI, history of respiratory disease, and smoking status

After controlling for the potentially confounding factors, the multivariate analysis revealed that the family history of all cancers and LC significantly increased the risk of MPLC (OR = 1.64, *P* = 0.009 and OR = 2.59, *P* = 0.000, respectively). LC was reported in 2.1% of mothers of the cases and 1.1% of mothers of the controls. The multivariate analysis identified obviously increased risk of MPLC (OR = 2.45, *P* = 0.000) associated with mothers influenced by LC history. The history of LC in mothers of the cases and controls also demonstrated a significant risk of MPLC associated with fathers influenced by LC history (OR = 1.55, *P* = 0.013). The findings of this study also suggested that LC in siblings was associated with a 1.66-fold (*P* = 0.001) increase in MPLC risk. LC cases diagnosed at a younger age (aged < 55 years) had an evidently increased risk of MPLC (OR = 2.39, *P* = 0.000). We classified the history of cancers. The data revealed that a family history of breast cancer was obviously associated with an increased risk of MPLC (OR = 1.49, *P* = 0.042). The history of gastrointestinal cancer also resulted in an elevated but not statistically significant risk of MPLC (OR = 0.97, *P* = 0.291).

Table 5 displays an analysis of the data by histology-specific MPLC risk in relation to family histories of cancers. An evident association with a family history of LC was found for male squamous carcinoma and male adenocarcinoma (OR = 1.59, p = 0.037 and OR = 1.64, p = 0.032, respectively). A positive association with a history of LC was only observed for female adenocarcinoma (OR = 2.23, p = 0.028). Conversely, there was no such positive association with a family history of cancers for any histological type of female LC.

**Table 5:** Odds ratios (OR) histological type of MPLC associated with family history of lung cancer in a first-degree relative

Histology	Cases n = 320	Controls n = 640	Crude OR (95% CI)	*p*	Adjusted^a^ OR (95% CI)	*p*
Family history of lung cancer in a first-degree relative						
Men	128	256				
Squamous carcinoma						
No	43	111	1.00		1.00	
Yes	23	21	1.72(1.26–2.61)	0.029	1.59(0.96–2.01)	0.037
Adenocarcinoma						
No	24	58	1.00		1.00	
Yes	14	18	1.84(1.34–2.81)	0.018	1.64(1.12–2.47)	0.032
Non-small cell carcinoma						
No	18	35	1.00		1.00	
Yes	6	15	1.99(1.14–3.01)	0.019	1.83(0.98–2.94)	0.065
Family history of lung cancer in a first-degree relative						
Women	192	384				
Squamous carcinoma						
No	43	102	1.00		1.00	
Yes	11	16	1.78(1.24–2.72)	0.039	1.21(0.96–1.91)	0.170
Adenocarcinoma						
No	62	172	1.00		1.00	
Yes	42	36	2.73(1.62–4.41)	0.019	2.23(1.42–3.57)	0.028
Non-small cell carcinoma						
No	29	59	1.00		1.00	
Yes	5	9	1.84(1.32–2.71)	0.023	1.43(1.01–2.02)	0.167

^a^Adjusted for age, dwelling, education, ETS exposure, BMI, history of respiratory disease, and smoking status

Table 6 reveals the relative risk for MPLC stratified by smoking status concerning a family history of cancer. The reference category was non-smokers with no family history of cancer and non-smokers with no family history of LC. In non-smokers, the proportion of a history of cancer and LC in the cases and controls was reported to be 27.9% vs. 15.1% of the history of cancer and 17.4% vs. 7.5% of the history of LC. In ever-smokers, the proportion of the history of cancer in the cases and controls was reported to be 32.9% vs. 21.1%. The proportion of history of LC in the cases and controls was reported to be 14.5% vs. 9.7%.

**Table 6:** Odds ratios (OR) for MPLC by smoking status and family history of lung cancer

	Cases n = 320	Controls n = 640	Crude OR (95% CI)	*p*	Adjusted^a^ OR (95% CI)	*p*
Non-smokers	86	332				
Family history of cancer						
No	62	282	1.00		1.00	
Yes	24	50	1.27(0.81–2.06)	0.184	0.91(0.66–1.16)	0.236
Family history of LC						
No	71	307	1.00		1.00	
Yes	15	25	2.51(2.16–3.64)	0.023	2.34(2.94–3.82)	0.031
Ever-smokers	234	308				
Family history of cancer						
No	157	243	3.58(2.76–5.01)	0.000	3.49(2.68–4.82)	0.000
Yes	77	65	4.11(3.74–6.63)	0.000	4.01(3.56–5.91)	0.000
Family history of LC						
No	200	278	3.68(2.87–5.18)	0.000	3.55(2.81–4.87)	0.000
Yes	34	30	6.74 (5.17–8.68)	0.000	6.49(5.06–8.19)	0.000

^a^ Adjusted for age, education, passive smoking, number of siblings

The results of the multivariate analysis revealed that an increased risk of MPLC was significantly associated with a family history of LC in non-smokers (OR = 2.34, *P* = 0.031) but not with a family history of cancer (OR = 0.91, *P* = 0.236).

The multivariate analysis indicated that smokers with a positive family history of cancer had a manifestly elevated risk of MPLC (OR = 4.01, *P* = 0.000). We also observed an evidently elevated risk for smokers with no family history (OR = 3.49, *P* = 0.000). In ever-smokers with a family history of LC, we found a manifest association with the risk of MPLC (OR = 6.49, *P* = 0.000). This positive association was also observed in ever-smokers with no family history of LC (OR = 3.55, *P* = 0.000). A synergistic effect of smoking status and family history of LC was observed in our analysis.

## Discussion

The incidence of multiple nodules in clinical practice has recently increased annually. Both incidences of squamous cell and small cell carcinoma have decreased, whereas that of adenocarcinoma has increased [14]. The risk of solitary primary LC was well established. Most studies have demonstrated an increased risk of overall LC for individuals with a family history of LC and tobacco [4, 15–17]. However, the patterns and risk factors of MPLC incidence are not straightforward [18, 19]. Our case-control study investigated the association between a family history of LC and MPLC risk among the Chinese population.

Our results demonstrated a positive association between a family history of total cancers and LC in first-degree relatives and risk of MPLC. This finding is consistent with the findings of solitary primary studies [20, 21]. However, this finding did not account for the genetic susceptibility of causing MPLC, as it may also result from the familial aggregation of shared lifestyle. Studies have demonstrated evidence of familial aggregation of LC [16, 22]. We believe that both shared environmental and genetic factors could contribute to the familial aggregation of MPLC.

Previous studies have reported that smoking is significantly associated with the risk of LC [20, 23]. Therefore, we collected additional information on all family members, including their smoking status. The reliability of our study’s results could be improved after adjusting for family members’ smoking habits. Our stratified analysis by smoking status (Table 6) also indicated that tobacco smoking had a significant effect on the risk of MPLC for both sexes. By contrast, the ratio of smoking in Chinese women is usually lower than that in men. These findings demonstrate that some inherited genes, such as cytochrome P450-related genes, may be involved in the role of tumor development [15, 24]. We found that almost a third of MPLCs were non-smokers, and the proportion of adenocarcinoma was higher than any other non-small cell carcinoma, indicating that the association between smoking and the risk of MPLC was weak. In Japan, a case-control study in LC also revealed that smoking plays a weak role compared with other histologic types [23]. In non-smokers, our results demonstrated that MPLCs were not significantly associated with a history of total cancers but were significantly associated with a history of LC. ETS exposure was higher in the MPLC group. Non-smokers with a history of LC may be able to eliminate inherited genes in modifying the susceptibility to tobacco carcinogenesis [15]. In LC prevention, Non-smokers should be notified about avoiding ETS exposure.

The findings of our study on the family history of other cancers and LC indicate some suggestion of familial risk of MPLC. History of breast cancer and LC was positively associated with MPLC, whereas no such associations were observed in gastrointestinal cancer. The role of early age in LC diagnosis is consistent with the findings of previous studies [20, 25]. This finding provided evidence of the familial risk of MPLC. Some studies have demonstrated that the early onset of LC may suggest families with a genetic predisposition to solitary primary LC [25]. Previous epidemiological studies of LC families have hypothesized that some genes interacting with smoking exposure contribute to the early onset of LC [26, 27]. There are some similarities in risk factors between breast cancer and solitary primary LC [28]. The history of LC among siblings was positively associated with MPLC. This finding also suggests that shared exposure to some environmental and residential factors among siblings may play roles in the development of MPLC. In previous studies of solitary primary LC, shared lifestyle and environmental tobacco exposure were found to play a crucial role in the development of LC [16, 29].

Consistent with previous studies of solitary primary LC, our results revealed an elevated risk for female adenocarcinoma. This result indicated that adenocarcinoma was more prevalent and associated with a family history of LC compared with other histologic subtypes [30]. Studies have demonstrated that the LC risk associated with smoking for adenocarcinoma is weaker than other histologic types [23]. One of our findings is consistent with a previous study by Xinjun et al. [31]. The proportion of MPLC with a family history of LC in females was higher (30.2%) than that in males (23.4%). We hypothesize that adenocarcinoma in females, especially in non-smokers, is associated with other risk factors, with familial aggregation being the most important one. Xin et al. conducted a case-control study of Chinese females in Singapore and found that family history of LC and adenocarcinoma was higher among non-smokers (OR = 2.39) than that among the overall population (OR = 1.9) [6]. One of important contributing risk factors to adenocarcinoma development in MPLC is family history of LC, especially in non-smoking females. In males, we also found that squamous cell carcinoma and adenocarcinoma were associated with family history of LC. A previous meta-analysis also reported the similar results [32]. However, further studies with multi-regions and larger population will be required to confirm our findings.

The risk of developing MPLC with a history of total cancers was not significant in non-smokers of our study; this may be due to the relatively small sample size of non-smokers, which affects the finding of significance. However, we observed a significantly increased risk of a family history of LC, indicating that the risk of non-smokers with MPLC is different from solitary primary LC. These results complemented the results of familiar aggregation of MPLC.

In ever-smokers, a family history of cancer and LC were significantly associated with MPLC. Jadwiga et al. (2009) ’s study (1058 women with histologically confirmed LC and 2116 healthy controls) demonstrated that family history of LC was a significant predictor of solitary primary LC risk and confirmed synergistic effect of smoking status and family history of LC in first-degree relatives [15]. In a study of 267 MPLCs from Sweden, Xinjun et al. found that patients with a family history of LC had a significantly increased risk [31]. These findings provide additional evidence for the familial and genetic risk of MPLC.

The present study has an advantage. This study is the first in an Asian population to investigate the association between family tumor history and MPLC risk. Our study has some limitations. This study was a hospital-based case-control study. The cases and controls selected from the same hospital or physical examination center were considered comparability. There might be some degree of selection bias. The information on family history was gathered through a questionnaire. Some information, such as ETS exposure and smoking, cannot be validated. There is the possibility of recall and information bias. By contrast, a previous review found that self-reporting in a study has sufficient credibility to be useful for epidemiological research [12]. Hence, it would not influence the final results of this study. The present study was performed at a single hospital in Fujian Medical University Union Hospital of China. To confirm our results, we should conduct additional research in other regions.

## Conclusions

In this case-control study, we confirmed the association of family history of LC with MPLC risk among the Asian population. Adenocarcinoma in females was prevalent and significantly associated with a family history of LC in risk of MPLC compared with other histologic subtypes. Smoking status and family history of LC have a synergistic effect on MPLC. These findings indicate that MPLC has familiar aggregation, and inherited genetic susceptibility may contribute to the development of MPLC. A family history of LC must be considered to prevent MPLC.

## Data Availability

The datasets used and/or analyzed during the current study are available from the corresponding author on reasonable request.

## Abbreviations

OR: odds ratios
MPLC: multiple primary lung cancers
LC: lung cancer
ETS: environmental tobacco smoke
